# Draft Genome Sequence of *Vibrio parahaemolyticus* VH3, Isolated from an Aquaculture Environment in Greece

**DOI:** 10.1128/genomeA.00731-15

**Published:** 2015-07-02

**Authors:** Daniel Castillo, Jin Woo Jun, Paul D’Alvise, Mathias Middelboe, Lone Gram, Siyang Liu, Pantelis Katharios

**Affiliations:** a Marine Biological Section, University of Copenhagen, Helsingør, Denmark; b Institute of Marine Biology, Biotechnology and Aquaculture, Hellenic Centre for Marine Research, Crete, Greece; c Department of Systems Biology, Technical University of Denmark, Copenhagen, Denmark; d BGI Europe A/S, Copenhagen, Denmark

## Abstract

*Vibrio parahaemolyticus* is an important foodborne pathogen responsible for gastroenteritis outbreaks globally. It has also been identified as an important pathogen in aquatic organisms. Here, we report a draft genome sequence of *V. parahaemolyticus*, strain VH3, isolated from farmed juvenile greater amberjack, *Seriola dumerili*, in Greece.

## GENOME ANNOUNCEMENT

*Vibrio parahaemolyticus* is one of the most important foodborne pathogens globally. Infections are almost exclusively associated with the consumption of raw or improperly cooked contaminated seafood (1, 2). Clinical characteristics include watery diarrhea, abdominal cramps, nausea, and fever (3, 4). However, *V. parahaemolyticus* infections are not exclusive to humans and have recently been demonstrated to be a major source of disease in aquaculture production (5, 6). Therefore, monitoring of this pathogenic *Vibrio* species in aquaculture environments is important for both the aquaculture industry and human health. In the present study, *V. parahaemolyticus* VH3 was isolated from the aquaculture facility of the Institute of Aquaculture of the Hellenic Centre for Marine Research (HCMR) at the port of Heraklion, Greece, during a vibriosis outbreak in juvenile greater amberjack.

Genomic DNA of *V. parahaemolyticus* VH3 was extracted using the QIAamp DNA minikit (Qiagen) according to the manufacturer’s protocol. The genome was sequenced by standard shotgun sequencing methods using a 454 GS-FLX Titanium sequencing system (Roche). The sequence data consisted of 5,600,000 reads (average length, 79 bp), providing 89-fold coverage. *De novo* assembly of the whole sequencing reads was performed with a GS De Novo Assembler. A total of 67 contigs, with a minimum length of 943 bp and a maximum length of 592,284 bp (*N*_50_, 187.7 kbp), were obtained. Annotation was performed by the NCBI Prokaryotic Genome Automatic Annotation Pipeline (PGAAP) (7). Additionally, the genome was screened for presence of specific genetic markers for *V. parahaemolyticus* (*tlh, tdh, trh*, *orf8*, and *toxRSnew*) (8), prophages (9), virulence factors (10), and antibiotic resistance genes (11).

The draft genome of *V. parahaemolyticus* VH3 was 4,955,051 bp in length, with a G+C composition of 45.3%. Genome annotation resulted in 4,338 coding sequences (CDS), 39 tRNAs, 37 pseudogenes, and 4 rRNAs. One phage-related sequence of 12.5 kb was found. Virulence factors related to adhesion (arylsulfatases and colonization factor Acfa), hemolysin, metalloproteases, and *Mycobacterium* virulence operon and multidrug resistance efflux pumps were found. Antibiotic resistant genes were detected for fluoroquinolones and tetracycline.

To the best of our knowledge, this is the first genome report of a *V. parahaemolyticus* strain isolated in an aquaculture system in Greece. The current sequence data generated here will contribute to the understanding of genome variability of *V. parahaemolyticus* isolates in future genomic studies.

### Nucleotide sequence accession numbers.

This whole-genome shotgun project has been deposited at GenBank under the accession number LCVL00000000. The version described in this paper is version LCVL01000000.

## References

[1] Daniels NA, MacKinnon L, Bishop R, Altekruse S, Ray B, Hammond RM, Thompson S, Wilson S, Bean NH, Griffin PM, Slutsker L 2000 *Vibrio parahaemolyticus* infections in the United States, 1973–1998. J Infect Dis 181:1661–1666. doi:10.1086/315459. 10823766

[2] Jun JW, Kim HJ, Yun SK, Chai JY, Park SC 2014 Eating oysters without risk of vibriosis: application of a bacteriophage against *Vibrio parahaemolyticus* in oysters. Int J Food Microbiol 188:31–35. doi:10.1016/j.ijfoodmicro.2014.07.007. 25086350

[3] Matsumoto C, Okuda J, Ishibashi M, Iwanaga M, Garg P, Rammamurthy T, Wong HC, Depaola A, Kim YB, Albert MJ, Nishibuchi M 2000 Pandemic spread of an O3:K6 clone of *Vibrio parahaemolyticus* and emergence of related strains evidenced by arbitrarily primed PCR and *toxRS* sequence analyses. J Clin Microbiol 38:578–585. 10655349 10.1128/jcm.38.2.578-585.2000 PMC86152

[4] Ottaviani D, Leoni F, Rocchegiani E, Santarelli S, Canonico C, Masini L, Ditrani V, Carraturo A 2008 First clinical report of pandemic *Vibrio parahaemolyticus* O3:K6 infection in Italy. J Clin Microbiol 46:2144–2145. doi:10.1128/JCM.00683-08. 18448694 PMC2446877

[5] Soto-Rodriguez SA, Gomez-Gil B, Lozano-Olvera R, Betancourt-Lozano M, Morales-Covarrubias MS 2015 Field and experimental evidence of *Vibrio parahaemolyticus* as the causative agent of acute hepatopancreatic necrosis disease of cultured shrimp (*Litopenaeus vannamei*) in northwestern Mexico. Appl Environ Microbiol 81:1689–1699. doi:10.1128/AEM.03610-14. 25548045 PMC4325143

[6] Tey YH, Jong KJ, Fen SY, Wong HC 2015 Occurrence of *Vibrio parahaemolyticus*, *Vibrio cholerae*, and *Vibrio vulnificus* in the aquacultural environments of Taiwan. J Food Prot 78:969–976. doi:10.4315/0362-028X.JFP-14-405. 25951392

[7] Tatusova T, DiCuccio M, Badretdin A, Chetvernin V, Ciufo S, Li W 2013 Prokaryotic genome annotation pipeline. National Center for Biotechnology Information, Bethesda, MD.

[8] García K, Torres R, Uribe P, Hernández C, Rioseco ML, Romero J, Espejo RT 2009 Dynamics of clinical and environmental *Vibrio parahaemolyticus* strains during seafood-related summer diarrhea outbreaks in southern Chile. Appl Environ Microbiol 75:7482–7487. doi:10.1128/AEM.01662-09. 19801458 PMC2786416

[9] Zhou Y, Liang Y, Lynch KH, Dennis JJ, Wishart DS 2011 PHAST: a fast phage search tool. Nucleic Acids Res 39:W347–W352. doi:10.1093/nar/gkr485. 21672955 PMC3125810

[10] Joensen KG, Scheutz F, Lund O, Hasman H, Kaas RS, Nielsen EM, Aarestrup FM 2014 Real-time whole-genome sequencing for routine typing, surveillance, and outbreak detection of verotoxigenic *Escherichia coli*. J Clin Microbiol 52:1501–1510. doi:10.1128/JCM.03617-13. 24574290 PMC3993690

[11] Zankari E, Hasman H, Cosentino S, Vestergaard M, Rasmussen S, Lund O, Aarestrup FM, Larsen MV 2012 Identification of acquired antimicrobial resistance genes. J Antimicrob Chemother 67:2640–2644. doi:10.1093/jac/dks261. 22782487 PMC3468078

